# SlicerArduino: A Bridge between Medical Imaging Platform and Microcontroller

**DOI:** 10.3390/bioengineering7030109

**Published:** 2020-09-11

**Authors:** Paolo Zaffino, Alessio Merola, Domenico Leuzzi, Virgilio Sabatino, Carlo Cosentino, Maria Francesca Spadea

**Affiliations:** Department of Clinical and Experimental Medicine, University “Magna Graecia” of Catanzaro, 88100 Catanzaro, Italy; merola@unicz.it (A.M.); domenico.leuzzi@studenti.unicz.it (D.L.); virgilio.sabatino@studenti.unicz.it (V.S.); carlo.cosentino@unicz.it (C.C.); mfspadea@unicz.it (M.F.S.)

**Keywords:** medical imaging platform, microcontroller, 3D Slicer, Arduino

## Abstract

Interaction between medical image platform and external environment is a desirable feature in several clinical, research, and educational scenarios. In this work, the integration between 3D Slicer package and Arduino board is introduced, enabling a simple and useful communication between the two software/hardware platforms. The open source extension, programmed in Python language, manages the connection process and offers a communication layer accessible from any point of the medical image suite infrastructure. Deep integration with 3D Slicer code environment is provided and a basic input–output mechanism accessible via GUI is also made available. To test the proposed extension, two exemplary use cases were implemented: (1) INPUT data to 3D Slicer, to navigate on basis of data detected by a distance sensor connected to the board, and (2) OUTPUT data from 3D Slicer, to control a servomotor on the basis of data computed through image process procedures. Both goals were achieved and quasi-real-time control was obtained without any lag or freeze, thus boosting the integration between 3D Slicer and Arduino. This integration can be easily obtained through the execution of few lines of Python code. In conclusion, SlicerArduino proved to be suitable for fast prototyping, basic input–output interaction, and educational purposes. The extension is not intended for mission-critical clinical tasks.

## 1. Introduction

Interaction between medical image platform and external environment has been always a desirable option in several applications. Intraoperative navigation [1,2,3], remote control [4,5], signal acquisition [6,7,8,9], and actuator control [10,11] are some examples. However, these kind of tasks usually require dedicated hardware and software, often expensive and mostly lacking of generalization for covering wide range of possible use cases.

3D Slicer (for the sake of simplicity, from now on it will be mentioned as “Slicer”) is an open source platform for medical image processing, analysis, and visualization, largely used for research and educational purposes [12,13,14]. It is written in C++ and Python, strongly relying on ITK [15], VTK [16], and QT [17] libraries. A dedicated Python interpreter is embedded into the platform. Slicer includes algorithms for image registration (rigid and deformable), image segmentation (manual, semi-automatic and automatic), volume rendering, mesh generation, and visualization. Due to the large amount of available extensions, it can be finely tailored to accomplish well-defined tasks. OpenIGTLink [18], in cooperation with Plus toolkit [19], is the principal interface for reliable and real-time navigation in Slicer. To achieve this, ad hoc setup of libraries and services is required, as well as dedicated and often expensive hardware. While it is the best choice for complex and critical tasks, users that need to interact with the external environment to execute simple operations could find this solution too complicated compared to their simple requirements.

Arduino is one of the most used microcontrollers worldwide [20,21]. It is open source, cheap, easy to program, and it can relies on a countless amount of external boards designed to extend its capabilities. Among the available hardware extensions there are connectivity adaptors (e.g., LAN and Bluetooth), servomotors, sensors (e.g., temperature, force, proximity, accelerometer, and gyroscope), high-power device drivers, cameras, and gesture recognition board. In addition to digital Input/Output (I/O) pins, multiple analog-to-digital converters are primitively embedded on the device, enabling an out-of-the-box acquisition of time continuous signals. Free and dedicated libraries are usually provided to interact with the chosen hardware extension. Several type of Arduino boards are available on the market, ranging from the smallest ones to more powerful devices featuring high number of I/O pins, digital–analogic conversion, and wireless on-board communication modules. Finally, it supports serial connection to share data with other devices (through two dedicated pins) and/or with a computer (via the USB port).

In light of this, the SlicerArduino module was designed and implemented to provide the user with a comfortable system to connect Slicer with the external environment via Arduino board. Due to its simplicity, it allows fast prototyping and basic I/O interaction, without claiming to be able to accomplish critical missions and high-complexity tasks. The proposed extension can be also used for educational purposes. The affordability of Arduino and its external boards, in cooperation with the open source Slicer capabilities, can represent a game changer for several applications. Data coming from/directed to Arduino are deeply integrated with the Slicer infrastructure, making it possible to easily interact with all the tools offered by the image processing and visualization suite.

## 2. Materials and Methods

### 2.1. General Extension Description

**Figure 1:**
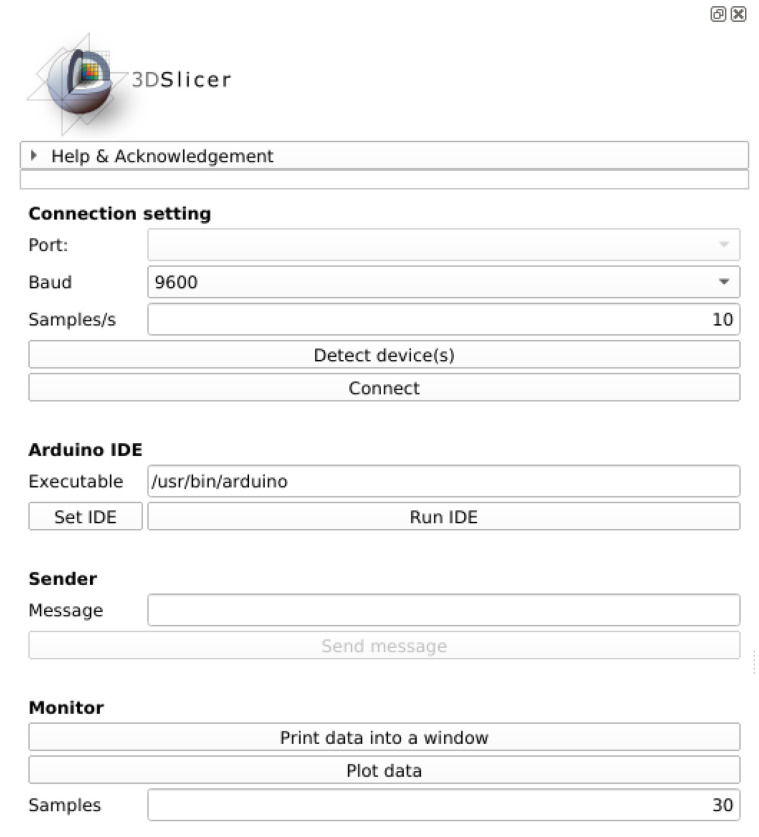
SlicerArduino Graphical User Interface (GUI).

SlicerArduino aims to provide a bidirectional link between the microcontroller and the medical image platform. The physical layer used to communicate is the USB port, natively embedded into all the Arduino boards. The entire extension is written in Python and, at a low level, it takes advantage of PySerial library [22] to manage the connection and the data stream to/from the board. The extension can be easily installed via the Slicer extension manager or, alternatively, it is possible to manually download the the source code [23] and add the folder to the extension path. A Graphical User Interface (GUI) was developed to allow the user to define connection parameters, to establish a link and to have a basic interaction with the microcontroller without writing code. In Figure 1, the extension GUI is depicted.

The GUI can be split in four sections, each of them dedicated to a specific aspect:

- **Connection setting:** In this section, it is possible to set the serial port for communicating with the Arduino board, the baud rate, and the sampling frequency used to interrogate the buffer. Once these parameters are defined, it is possible to connect/disconnect the device. The list of the available serial ports is dynamically created by inspecting the connected hardware.
- **Arduino IDE:** As it could be necessary to reprogram the board, the Arduino IDE can be run directly from Slicer. The extension automatically searches for the IDE executable in the system path or, alternatively, it can be manually set. Once the path is defined, it will be made persistent until a new choice is made.
- **Sender:** If a connection is established, by using this section is possible to send text string to the board.
- **Monitor:** Data coming from the board can be inspected in real-time. If the data stream contains characters and/or numbers, they can be shown in a dedicated window. If the data stream is made only of numbers, they can be plotted by taking advantage of the Slicer plotting infrastructure. The amount of samples to plot can be defined by the user. Monitoring the stream does not interfere with the other tasks that use Arduino data.

**Figure 2:**
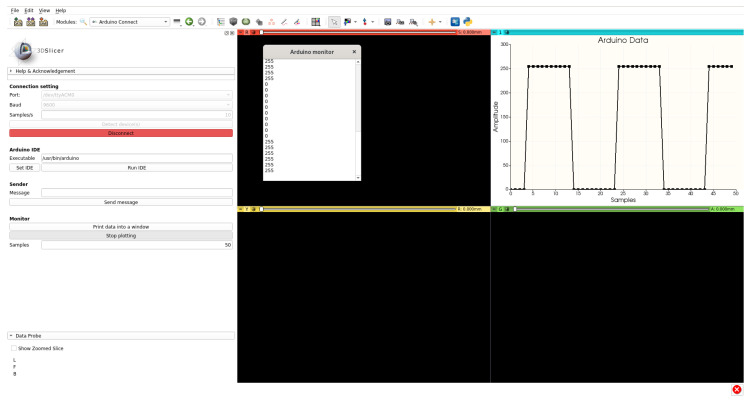
Data stream visualization coming from an established hardware connection.

In Figure 2, an example of the full SlicerArduino GUI interacting with Slicer is reported. In this case, Arduino sends a square signal (ten 0 values followed by ten 255 values) and, after the connection has been established, the data stream is both plotted and shown into the monitor window.

### 2.2. Slicer Integration

A key point of the entire project is the deep integration between Slicer infrastructure and data coming from/directed to Arduino. Slicer massively uses vtkMRMLNode objects to store and represent different type of data (volumes, transformations, and segmentations). The best way to incorporate data coming from the external environment and to interact with the Slicer environment is to exploit the advantages arising from the methods that nodes offer. For this reason the extension creates a dedicated node, where the value read from the board is stored into a specific parameter. As SlicerArduino serves as a base layer for developing additional code, it is necessary to notify the entire software environment that new data have arrived from the board and that it is stored into the node. This is achieved by taking advantage of the notify/observer mechanism already implemented into the vtkMRMLNode and largely used in Slicer. As a result, from any point of the Slicer infrastructure (including extensions and the embedded Python console), it is sufficient to observe the Arduino node to execute a specific method when a new value is received from the board and stored into the given parameter. There are no limitations about the maximum number of observers, so the same data can be simultaneously accessed by multiple applications (e.g., the plotter and the monitor shown in Figure 2). Finally, to send data from Slicer to the board, the sender method of the object instantiated by the SlicerArduino GUI can be directly executed, without using the vtkMRMLNode instance.

**Figure 3:**
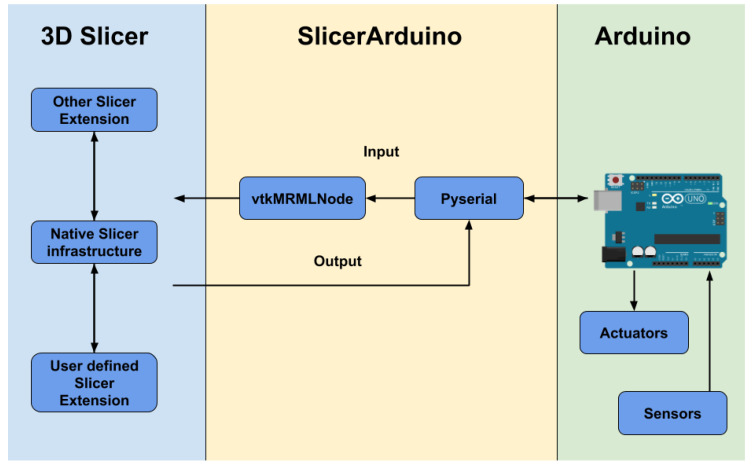
Graphical concept of SlicerArduino extension.

A graphical concept of the extension is shown in Figure 3.

### 2.3. Exemplary Use Cases

In order to test the functionality and performance of ArduinoSlicer, two exemplary use cases were implemented: one to receive signals from the board, and one to send instruction to the microcontroller. In both experiments, Slicer was ran on a GNU/Linux laptop and an Arduino UNO R3 was used. For the Slicer side the code was written in Python, Arduino was programmed by its C-like language.

#### 2.3.1. From External Environment to Medical Imaging Platform

**Figure 4:**
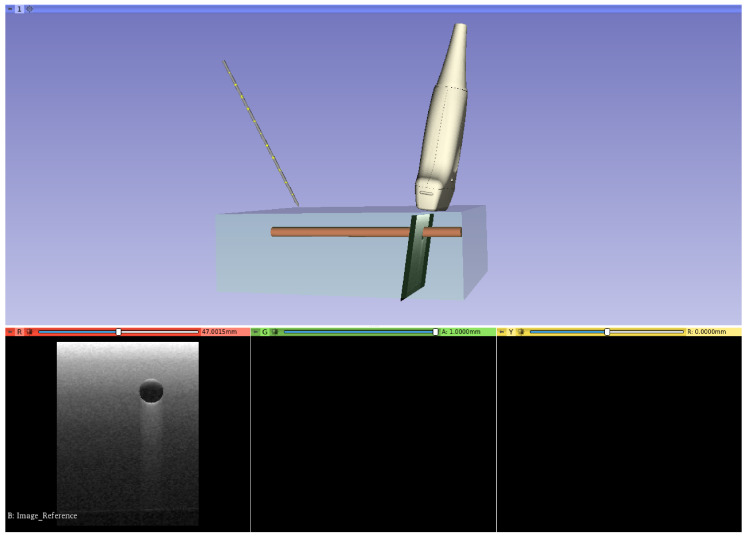
Simulation of an ultrasound guided procedure. The aim was to move the probe according to the data coming from a distance sensor.

In the first experiment, a Slicer scene mimicking an ultrasound guided procedure was loaded and a distance sensor (SHARP 2Y0A21) was connected to Arduino. The goal was to apply, in quasi-real-time, a translation to the probe on basis of the data coming from a hand moving closer or further from the sensor. In this way, the deep integration achievable between Slicer and Arduino was highlighted and stressed. A Slicer transformation, in fact, can be applied to any volume, model, or segmentation. In light of this, it was sufficient to use the value received from the microcontroller to edit the transformation and, as a consequence, move the model. The anatomical image was updated as well. Figure 4 shows this use case, while the Python code used for the task is reported in code block (Listing 1).

#### 2.3.2. From Medical Imaging Platform to External Environment

**Figure 5:**
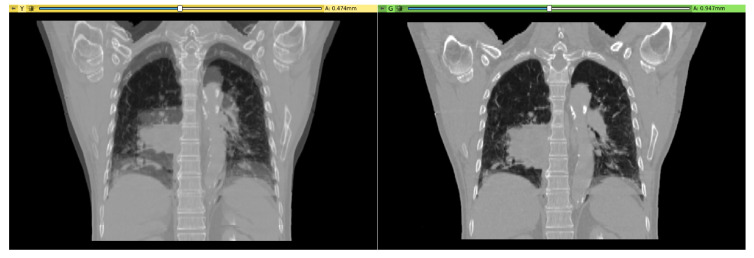
Computed tomography (CT) scans shown in overlay mode. In the left panel, before registration, it is possible to see the misalignment along the z axis. In the right panel, after linear registration, the displacement was accurately recovered.

In the second experiment, two chest computed tomography (CT) scans were loaded in Slicer and a linear servomotor (Actuonix L16 Actuator 50 mm) was connected to Arduino. By using the Slicer infrastructure, the CT images were rigidly registered (only a displacement along the Z-axis was required to align the volumes, as reported in Figure 5) and the servomotor arm was moved by the same quantity found for the Z translation. Moreover, in this case it was possible to easily control an external device (in this instance a motor) on the basis of a parameter typical of an image processing workflow. The basic idea was to mimic the patient’s position correction process executed before radiotherapy session by moving the machine couch. The code developed for Slicer is reported into code block (Listing 2).

## 3. Results

Both experiment goals where successfully achieved. In the first case, the hand movement smoothly controlled the probe translation in Slicer; in the second case, the servomotor moved accordingly to the computed transformation. In this regard, to provide the reader with a better insight into the test results, a video recording is available as supplementary materials.

The source code required to accomplish the exemplary tasks was very short and simple, and proved the simplicity of interaction between hardware and software platforms. Integration between Slicer environment and data coming from/to the board was direct and transparent.

During the first experiment, a smooth trajectory tracking was achieved, suggesting the capability to also execute tasks where time response is an important parameter; during the second experiment, the servomotor took 3 s to reach its final position (displacement equal to 25 mm). No visual severe Slicer lag, freeze, or crash occurred, proving that the pooling thread does not interfere with the main process.

Finally, connection stress tests were also conducted. One-hundred messages were successfully sent from Slicer to Arduino in 192 ms. Another test was to measure the time required for a message to travel back and forward the board, passing through Slicer infrastructure. This was quantified for two different polling frequencies, 50 Hz and 100 Hz, and the experiment was repeated 20 times for each frequency. Results are reported in Table 1.

## 4. Discussion

In the last years, important progress has been made in the development of more powerful and accessible image processing platforms (e.g., the open source project “3D Slicer”) and microcontrollers (like Arduino). In the light of this, SlicerArduino was implemented to provide a simple link between Slicer and the external environment, via Arduino board. The main applications of this plugin are about fast prototyping, basic input–output tasks and educational purposes.

Any clock signal is natively provided to interface multiple devices. However, this does not exclude the implementation of a custom synchronization mechanism by the user. We are aware that the absence of a predefined sync strategy could represent a disadvantage for certain applications. However, it is important to highlight that the proposed extension was not designed for mission-critical and high-complexity scenarios (e.g., connection with a commercial surgical navigation platform and/or multiple devices). In such cases, Slicer already has well-tested and powerful solutions [18,19]. Rather, the proposed extension aims to support users that want to develop from scratch their own custom solution.

The strength of the proposed work is about simplicity of communication and accessibility of both hardware and software. In fact, Slicer and its extensions are free, open source, and easily customizable; the Arduino microcontroller and its countless additional boards, sensors, and actuators are cheap and can be adapted to a wide range of cases. As a result, joining Slicer and Arduino may enable a large number of potential applications.

In the proposed experiments, a single sensor/actuator was used, but the combination of multiple input/output devices can lead to an advanced interaction. It is important to highlight that both of the proposed experiments served only as a proof of bidirectional communication between the medical image suite and the external environment.

Being Slicer binaries available for multiple platforms (Windows, Mac OSX, and GNU/Linux), no restrictions about the preferred operating system exist. The proposed plug-in can be installed via the Slicer extension manager or by manually downloading the source code, independently of the used platform.

Due to the homogeneous Arduino environment, the entire family of microcontrollers can be interfaced with Slicer via the proposed bridge. In this way, the user will choose the best hardware solution for his task (physical size, maximum current per pin, number of I/O pins, memory, etc.) without the need to edit the Slicer code. In addition to managing the connection part, a basic sender and an input monitor/plotter are also available via GUI.

A fundamental point of the entire project is the deep integration between data coming from/directed to the board and the Slicer environment. Data, in fact, are not just read, but stored into a dedicated vtkMRMLNode, one of Slicer’s pillars. As a result, interaction with other nodes is immediate and simple. The raw data, in fact, would be almost useless and it would not be possible to take advantage of the full potentialities of Slicer. The observe mechanism guarantees the possibility to notify to the entire environment the arrival of a new data from the microcontroller, allowing to write code that can react in quasi-real-time to the data stream. In addition, this makes it possible to access the data from different instances, simultaneously. No evident lag or freeze of the main process that could compromise the comfortable interaction between internal and external environments occurred during the tests. In this paper, the definition “quasi-real-time” was used, even if the amount of time required to acquire/send the data was extremely low (see Table 1). The adequacy of this parameter depends on the specific task the user wants to achieve.

As proved by the proposed experiments, the coding effort needed for the user to make Slicer interacting with Arduino (and vice versa) is minimal. If necessary, the input data stream can be filtered and conditioned distributing the computational effort between Arduino and Slicer. It is important to highlight that is also possible to interactively communicate with the board by using the embedded Python console.

Due to the plethora of possible sensors/actuators/boards and the way of combining them for accomplishing a well-defined task, it was not possible to embed in the extension a more specific interface (e.g., preconfigured options) for an out-of-the-box support of a specific device.

Both Slicer [24] and Arduino have a very active community and a complete documentation, which helps beginners to improve their knowledge and to accomplish the task. SlicerArduino’s documentation is also available on the project webpage [25].

The main current limitation regards the possibility to connect just a single board, while future efforts will be aimed toward adding a Bluetooth connection and testing the compatibility with microcontrollers different from Arduino family (e.g., TexasInstrument and STMicroelectronics).

## 5. Conclusions

For the fist time, a bridge between one of the most used medical image processing platform (3D Slicer) and a popular microcontroller family (Arduino) was developed. A simple and powerful interaction between them can be achieved, enabling a wide range of possible applications. The main envisioned applications are related to fast prototyping, basic I/O tasks, and educational purposes in the medical image field.

## Figures and Tables

**Listing 1:**
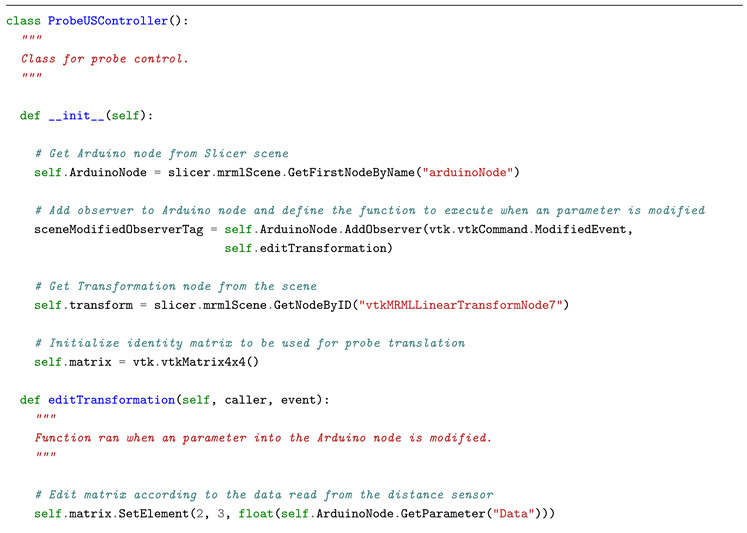

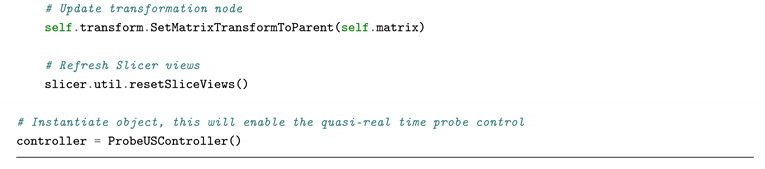
Python code developed for moving ultrasound probe according to data coming from distance sensor. Since the class is observing the Arduino node, when a new value is read the function that edits the linear transformation is executed.

**Listing 2:**
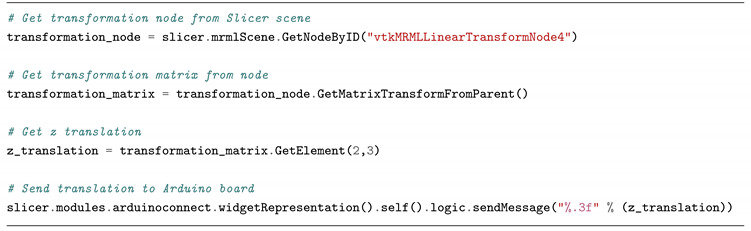
Python code for controlling a servomotor according to a translation identified by the alignment of two CT scans.

**Table 1:** Time needed for a sample to travel back and forward the board via Slicer (at 9600 baud).

Polling Frequency (Hz)	Mean (ms)	Standard Deviation (ms)
50	20.0	2.4
100	17.4	1.7

## Supplementary Materials

The video is available online at https://www.mdpi.com/2306-5354/7/3/109/s1.
